# Supplementary material for: A single-blind, randomised control trial on the effectiveness of a structured multi component training module for family caregiver of persons with Parkinson’s disease: A study protocol
Source: PLoS One. 2024 Nov 11;19(11):e0309648. doi: 10.1371/journal.pone.0309648 (PMC11554143; doi:10.1371/journal.pone.0309648)
Supplement: S1 File — (DOCX) [file pone.0309648.s002.docx]

**DEVELOPMENT OF STRUCTURED MULTI COMPONENT TRAINING MODULE FOR FAMILY CAREGIVER OF PERSONS WITH PARKINSON’S DISEASE**

**Researchers:**

**Assoc. Prof. Dr. Nor Azlin Mohd Nordin Prof Dr Norlinah Mohamed Ibrahim Prof Assoc. Prof Dr Aniza Ismail**

**Dr Hanif Farhan Mohd Rasdi Sharmila GK Pillai**

**Nur Izyan Mohd Amin**

**Faculty of Health Sciences & Faculty of Medicine Universiti Kebangsaan Malaysia.**

**2022**

1. **Background**

Parkinson’s disease (PD) is a progressive neurodegenerative disorder characterized by motor and non-motor features. The disease progression is contributed by progressive denervation and neuro-degeneration of various areas in the brain and peripheral nervous system (Kouli et al. 2018; Poewe & Mahlknect, 2009). Clinically, this is demonstrated by increasing severity of motor features for example postural instability and freezing of gait following the evolution of poor responsiveness towards PD medications. Further, a wide range of non-motor disorders may develop and contribute significantly to disability experienced by PD patients (Kouli et al. 2018). Parkinson's disease is the fastest developing of the neurological illnesses that are currently the major cause of disability in the world. As the population ages and life expectancy rises, so does the cost of healthcare. Statistics of people suffering from PD will continue to increase, as does the duration of condition, resulting in greater PD sufferers in advanced stages (Ray Dorsey et al. 2018).

The global prevalence of PD based on the 2016 statistics is 6.1 million (Ray Dorsey et al. 2018). The global burden of PD in the same year is high, reported as 3.2 million disability adjusted life years (DALYs) with greater than 200,000 reported deaths. The burden of PD worldwide is expected to rise due to increased life expectancy of the ageing population, which increases the necessity for extended care (Ray Dorsey et al. 2018). The prevalence of PD in Malaysia as of 2016 statistics are 19 586 with death count as 514. The age-standardized rate of DALYs in Malaysia is estimated as 9694 with 19.1% increase from 1991(Ray Dorsey et al. 2018). This data is alarming as it is further reinforced by a report published by the Department of Statistics Malaysia in 2018, the number of people with Parkinson's disease in Malaysia is anticipated to increase fivefold by 2040, from an estimated 20,000 to 120,000 (Hassandarvish, 2019).

Mobility is defined as the capacity of people to transport from one place to another, in order to participate in the activities of daily living (ADL) in various settings and this is greatly affected in persons with PD. Limitation in mobility further causes devaluation amongst PD patients, followed by sadness and frustration (Bouca-Machado et al. 2020). A recent study by Zhao and colleagues (2020), reported in most dimensions, persons with PD demonstrated lower quality of life (QoL) than healthy controls, particularly in physical function and mental health. Given the deleterious impact of poor QoL on daily living and functional results, effective approaches to increase QoL in this population should be developed.

Similar trend is observed in Malaysia with the nonmotor aspect of PD greatly contributing to the decline in QoL in persons with PD as measured by Parkinson Disease Questionnaire-39 (PDQ-39 QoL). The caregivers' burden was also influenced by PDQ-39 QoL dimensions such as "mobility," "activities of daily life," "emotional well-being," and "stigma" (Rajiah et al. 2017). The progressive nature of PD with increasing disabilities imposes a significant burden to the patient's family, being the main caregiver. Literature documented high occurrences of depression and anxiety among the caregivers of persons with PD, which corresponds on the experienced caregiving burden (Mosley et al. 2017).

Further, PD poses a substantial economic burden. It is estimated that the cost of care in Asia for a person with PD range from 649 and 9544 Euro (€), with patients in home care had annual direct expenditures of 445 € per person while hospitalized PD persons had annual direct costs of 9,544 € per person (Maresove, Klimova & Kuca, 2017). In China, the average yearly cost per persons with PD is $3,225.94, with direct and indirect expenditures of $2,503.46 and $722.48, respectively. Estimated costs for persons with PD for surgery is $556.27, with appointment fees of $44.67, prescription medicine of $605.67, hospitalization costs $460.29, auxiliary examination costs $71.03, transportation costs $35.64, special equipment costs $10.39 and formal care costs

$719.50. Surgical therapy, dopamine agonist, and levodopa prices also contributes to the grand total (Yang & Chen, 2017). A recent study reported that family caregivers of persons with PD patients also experience income loss and are burdened with high cost of care throughout the care process (Martinez Martin et al. 2019).

Optimum PD management requires a multidisciplinary approach and participation from various health care professionals to address the multiple needs of the patient (van de Marck et al. 2009). However, family caregivers play the utmost vital role as they determine the final institutionalization and quality of care of the patient (Gultekin et al. 2017). This justifies the enforcement of family education and training from the early stage of PD to better equip family caregivers with the required knowledge and care skills as the disease progresses. Structured and comprehensive training program, consisting of all aspects of care, from multidisciplinary approach would enable the family to be better prepared for their role and minimize emotional and physical burden.

A review of the literature revealed that there are few studies that explored family training programmes. A study by Villasenor et al. (2020), examined the connections between family dynamics and sense of coherence, among PD family caregivers in Mexico and the United States.

The study concluded family dynamics play an important role in PD caregiver coping which improves caregiver comprehension, manageability, and significance. The study however, is a correlational study which provides preliminary evidence that a sense of coherence develops in similar ways across these cultures, and strengthens the needs of intervention focusing on family dynamics. A recent randomized controlled trial (RCT) by Bagheri et al. (2019) investigated the effect of a family-centered empowerment model on the burden of care among the caregivers of the older adults with PD. The authors indicated such a model will decrease the burden of care while improving the role of caregivers. The study further provides recommendations that carers of patients with chronic conditions should participate in family-centered empowerment initiatives. The family-centered empowerment model by Bagheri and co-researchers (2019), however focuses on patient education rather than therapy without incorporating any measures evaluating the effectiveness of such therapy on functional outcomes of persons with PD.

A quasi experimental study (Navratha Sanche et al. 2019) comparing the effects of a nine- week psychoeducational intervention and five-week education program on people with PD and their informal caregivers' QoL, psychosocial adjustment, and coping showed significant differences between groups for coping skills of patients and caregiver’s psychosocial adjustments. However, the intervention in the study by Navratha Sanche and co-researchers (2019) targeting both the persons with PD patients and caregivers are of psychosocial education and not physically related to coping skills during physical limitation of the patient. There were also no outcome measures evaluating the functional outcome of the PD patient, similar to the study by Bagheri et al. (2019).

A review (Kessler & Liddy, 2017) identifying the characteristics of self-management programs for persons with PD and the evidence for their effectiveness, identified 18 interventions. Only four of 18 interventions included caregivers or family members, thus indicating the lack of studies targeting family oriented intervention to address the need of persons with PD. Another developed program by Chaplin, Hazan & Wilson (2015), titled Hertfordshire Neurological Services Self-Management Programme is aimed to provide a module based program for neurological patients including persons with PD. The theoretical underpinnings of this module is social cognitive theory and self-regulation model, hence the module is based on education - problem solving goals minus the focus on the physical functions outcome improvement. Despite inclusion of caregivers, the module is not exclusive to PD but also stroke and multiple sclerosis.

A past study by A’Campo et al. (2010) evaluated a psychosocial education program named Patient Education Program for Parkinson (PEPP), which was developed by a consortium of experts from several European countries, with inputs from PD patients and their caregivers. The program aimed to assist PD patients and caregivers to adapt to the life changes triggered by PD. A total of 137 caregivers and 151 PD patients participated in the 8-week program. The researchers found that the education program was useful in improving the knowledge level and quality of life (QoL) of participants. A’campo et al. (2010) also proved the feasibility of such intervention to be executed in various countries. The drawback of the study is, similar to Chaplin, Hazan & Wilson (2015), there is no therapy based education program, as the intervention focuses on psychosocial without physical elements plus no functional outcomes were measured. The feasibility of such module is also limited to European countries.

While another study by Guo and colleagues (2009) reported that group education with personal rehabilitation was beneficial and fulfilled the long-term care needs of the PD population, it is not targeted to the family. The researchers included input from persons with PD and their caregivers in the formation of the educational program developed by a multidisciplinary team led by a neurologist. The program was well accepted by the caregivers who perceived it as useful in improving their knowledge, however the intervention is self-centered. Another RCT by Lewis et al. (2009) conducted a study to describe the Stress-Busting Program (SBP) for family caregivers and its effectiveness with three phases of intervention. The study reported significant decrease in perceived stress, depression, subjective caregiver burden, anxiety, and anger or hostility among caregivers. Caregivers also had improved general health, vitality, social function, and mental health scores. Similar to Guo et al. (2009), this intervention does not target to help caregivers cope with the physical limitation of PD patients, rather focused on reducing stress of caregivers.

All the programs published in reviewed studies (Hellqivst et al. 2020; Bagheri et al. 2019; Navratha Sanche et al. 2019; Kessler & Liddy, 2017; Chaplin, Hazan & Wilson, 2015; A’ Campo et al. 2010; Guo et al. 2009; Lewis et al. 2009) lack comprehensiveness as they delivered mainly education on PD and its care, without care skills training such as rehabilitation on the functional limitation. The programs' clinical and cost effectiveness were also not assessed, warranting further research to evaluate these aspects. With the expected rise in the number of PD patients in Malaysia (Hassandarvish, 2019), there will be greater demand on medical care and rehabilitation. However, despite this projection, local research on care and rehabilitation remains limited, with no research available to date on family training programs for the PD population in Malaysia. Two studies reported significant burden among caregivers of Malaysian PD population,

which is associated with the patients' advancing age, later stage of illness, presence of emotional disturbance and longer duration of care (Rosdinom et al. 2011, Rajiah et al. 2017). A recent local study (Vasanthi & Choo, 2021) reported that exercise in the presence of family and friends is a great facilitator for exercise. Similarly, Mosley et al. (2017) found that interventions by trained family caregivers at home assist in improving functionality and eventually quality of life, hence need to be included in the overall care program for PD. This justifies the need for a structured multi-component, sustainable and cost effective family training program for persons with PD. The summary of the literature review is attached in the table 1 literature review matrix.

## PROBLEM STATEMENT

Family plays a vital role in the care of persons with Parkinson's disease. Due to the nature of the disease being chronic and progressive, with accompanying disabilities which worsen over time, care for this population is a significantly challenging task (van de Marck et al. 2009). The caregivers often experience long-term emotional and physical burden across all stages of the patient’s disease, which eventually threatens their well-being and quality of life. These, in return may reduce the quality of care given to the patient they cared for and consequently, affect the patient’s functional level and general health (Jenkinson et al. 2012). Therefore, caregivers’ wellbeing deserves attention in minimizing these consequences. To address these concerns, education and training to the family of persons with PD is important to equip them with adequate knowledge and care skills particularly as the disease progresses (Gultekin et al. 2017). Structured and comprehensive training programs would also enable the family to be better prepared for their role as caregivers and minimize emotional and physical burden, and these may result in improved QoL of the persons with PD and the caregivers. This is conceptualized in a framework as attached in appendix 1.

Currently, a few family education programs for people with PD are available as documented in the studies (Hellqivst et al. 2020; Bagheri et al. 2019; Navratha Sanche et al. 2019; Kessler & Liddy, 2017; Chaplin, Hazan & Wilson, 2015; A’ Campo et al. 2010; Guo et al. 2009; Lewis et al. 2009). However, the available programs are not comprehensive and focus mainly on knowledge regarding PD and its care, without the inclusion of care skills training. The programs have also not been evaluated for clinical effectiveness of variables such as functions and mobility plus the programs' sustainability and cost effectiveness remains questionable due to lack of follow up data. Due to these, more research that looks into developing and testing family training programs are needed.

In Malaysia, no research data is found to date on any structured family training program and its benefits on persons with PD. Available programs which are published in overseas studies, other than being less comprehensive, may not be suitable to be used in Malaysia due to the differences in care settings and organization of family life. A few local studies involving caregivers of the PD population reported significant perceived burden which was multi-factorial. However, no specific evidence-based program has been developed until now to overcome these caregivers' burden. Owing to the increase in the prevalence of PD in Malaysia as a result of population ageing, and the care burden that it heightened, it is warranted that an effective training program is to be developed and utilized towards enhancing the well-being of the person with PD and their family caregivers in this country. It is also emphasized in a recent study (Lidstone, Bayley & Lang, 2020) on the difficulty to evaluate the structure and efficacy of care models as these are not effectively reflected in clinical trial.

## NOVELTY AND RESEARCH SIGNIFICANCE

Structured, comprehensive family-training module has not been developed for persons with PD in Malaysia, and thus will be valuable for the local PD population and their family caregivers. This study will augment knowledge into the existing literature related to PD management and have implications on PD management of PD as both the persons with PD and their family caregivers are well prepared from Stage I or II of the disease. PD is a progressive disease, thus the home based clinical management is more apt for a smoother transition to palliative care later. Family assisted care training is crucial in PD as there is a shift of care from institution to community. There is no such study in Malaysia till date evaluating the effectiveness of family-assisted care training in persons with PD at home-based setting and therefore this study will provide crucial information on the feasibility information and the effectiveness. The evaluation of cost- effectiveness of training family as caregivers to implement care for patients with PD has not been attempted to date. Considering that PD is a chronic condition that requires long term care, evaluation of clinical efficacy as well as costs of a training program for caregivers are important to help reduce the disease burden. Caregiver burden has proven to reduce the quality of care provided to patients thus affecting the patient’s health and caregivers’ ability to provide care determines institutional placement. Therefore, development of this module which may be effective in improving function and QoL of PD persons through family assisted care. Further, providing rehabilitation from home is cost effective than institutionalization. It is important to train caregivers as Kalra and colleagues (2004) reported that training caregivers reduces burden and cost while improving outcomes for both patient and carers.

Table 1 Literature Review Matrix

| No. | Author and year | Aim | Methods | Intervention | Results and conclusion |
| --- | --- | --- | --- | --- | --- |
| 1. | Hellqivst et al. 2020 | To assess the outcomes of the Swedish  National Parkinson School (NPS) | Design: A quasi experimental study  Sample: 91 persons with PD patients and 55 family caregivers.  Outcome measures:   - Health status: PDQ-8 - Health valuation: EQ-5D - Health education impact: HeiQ - Life satisfaction: LiSat- 11 - Fatigue: PFS-16 - Caregiver burden: ZBI | - Programme based on principles of cognitive behavioral therapy and was developed based on the PEPP. - NPS includes the promotion of awareness of thoughts, feelings, and actions in relation to the impact of disease on daily life. - NPS also introduces techniques of self-monitoring in order to deliver the knowledge and tools in managing PD. | - Persons with PD reported improved health as indicated by both PDQ-8 (*p* = 0*.*028) and EQ5D (*p* = 0*.*023) scores following enrollment into NPS. - Improvements were also observed in the constructive attitudes and approaches (*P=* 0*.*003) and skill and technique acquisition (*P* < 0*.*001) of the HeiQ. |
| 2. | Lyons et al. 2020 | To explore the health benefits, self- management behaviors, illness communication  for partners in existing community-based self-management workshop for PD | Design: Quasi experimental  Sample: intervention group and control group consist of persons with PD and their caregiver (N = 39)  Outcome measure:   1. SF-36 2. CES-D 3. MCSI 4. Evaluation questionnaire CDSMP- Curriculum 5. Active engagement und protective buffering (VAS Scale) | Strive to thrive: Self-Management for Parkinson’s Disease   1. Self-management skills like monitoring, taking action, problem- solving, decision-making and evaluating results 2. Exercises (not further described), relaxation techniques 3. Six weeks of Chronic Disease Self-Management Program plus one week based on PD-specific content in group intervention format. | Preliminary data (*n* = 39 couples) reported couples in the intervention group had better engagement in mental relaxation techniques at 7 weeks compared to control condition.  Small effects were observed for increases in aerobic activity and mental relaxation for persons with PD, increases in strength-based tasks and self-efficacy for partners, declines in depressive symptoms for partners, and reduction in protective buffering for both adults with PD and their respective couples. |

| 3. | Bagheri et al. 2019 | This study  investigated the effect of a family- centered empowerment model on the burden of care among the  caregivers of the elderly with PD. | Design: RCT  Sample: 60 Caregivers and elderly with PD  Outcome measure: ZBI | Family-centered empowerment model consisted of four steps: Step 1: perceived threat  Step 2: problem-solving  Step 3: Educational participation Step 4: Evaluation  Intervention details: Three 10- persons groups in four- 45 minute sessions 2 days a week for 2 weeks. | Reduction in caregiving burden and improvement in the role of caregivers are observed.  Recommended to implement family- centered empowerment programs for the caregivers of persons with chronic diseases. |
| --- | --- | --- | --- | --- | --- |
| 4. | Navratha Sanche et al. 2019 | To evaluate the effects of a psychoeducational intervention compared with an education programme to  strengthen QoL, psychosocial adjustment, plus coping in people with PD and their caregivers | Design: Quasi Experimental Study  Sample: A total of 140 people with PD and 127 informal caregivers  Outcome measure: PDQ- 39, SQLC, PAIS-SR, The BRIEF COPE Scale. | The experimental group: nine week psychoeducational intervention  Control group: five week education programme. | Significant differences are observed between experimental and control groups for coping skills of patients and caregiver’s psychosocial adjustments.  Patients’ QOL and coping skills improved while the caregiver's coping skill and psychosocial adjustments improved following the intervention. |

| 5. | Kessler & Liddy, 2017 | To identify the characteristics of self-management programs for persons with PD and the evidence for their effectiveness. | Design: integrative literature review  Sample: Eighteen intervention studies. | 11 interventions were group based withy 1 and half hours per week for six to eight weeks delivered in person.  7 interventions were delivered on an individual basis, despite majority provided face to face, 2 intervention were delivered virtually.  Of the 18 interventions included in the study, only 16 were designed specifically for PD persons while remaining generally offered to persons diagnosed with chronic neurological disease.  Out of 18 interventions, only 4 interventions included caregivers or family members.  Key components of self-management support are education, problem- solving and goal setting. | |
| --- | --- | --- | --- | --- | --- |
| 6. | Chaplin, Hazan & Wilson, 2015 | To evaluate the Hertfordshire Neurological Services Self- Management Programme | Design: Program Evaluation survey post intervention  Sample: Stroke, PD, Multiple Sclerosis  Outcome measures: N/A | Intervention consist of following:  Module 1 – ‘What is self- management: pros and cons’ (all conditions, for service users and carers),  Module 2 – ‘Living Well’ (all conditions, for service users only)  Module 3 – Condition-specific information (for service users only). | Participant’s states expectations met from the modules.  Discussions and hearing other’s experiences was beneficial outcome of the course.  Suggestions to involve the caregiver in all modules |

| 7. | A’ Campo et al. 2010 | To evaluate the Effectiveness of the PEPP | Design: RCT  Sample: 65 PD patients and 47 caregivers  Outcome Measure:  The impact of psychosocial problems: BELA-P-k and BELA-A-k.  QoL patients: PDQ-39.  QoL caregivers: EQ-5D Depression: SDS. | PEPP is a standardized psychosocial intervention. The intervention consisted of eight weekly sessions of 90-minute duration.  8 sessions of PPEP; information, self-monitoring, health promotion, stress management, management of anxiety and depression + caregiver challenges, social competence, social support, evaluation | A significant effect for the caregivers on psychosocial problems & need help was observed and a trend for significance for patients’ QoL.  Persons with PD and caregivers’ mood improved significantly after each session. |
| --- | --- | --- | --- | --- | --- |
| 8. | A’ Campo et al. 2010  (2) | To undertake a formative evaluation of a standardized psychosocial education program for persons with PD and their respective caregivers | Design: Formative  evaluation of an experimental study  Sample: 17 caregivers and 151 PD patients participated in the 8-week program in separate groups.  Outcome measure: The impact of psychosocial problems: BELA-P-k and BELA-A-k.  QoL patients: PDQ-39.  QoL caregivers: EQ-5D Depression: SDS | Similar to above.  The program consisted of eight weekly sessions of ninety minutes. Groups consisted of 4–7 participants. | The program was feasible to run in the different countries. |

| 9. | Guo et al. 2009 | To evaluate the effect of a group education program with personal rehabilitation for persons with PD | Design: RCT with a pre- test/post-test quasi- experimental design.  Sample: 44 patients with PD Outcome measure:  PDQ-39, UPDRS, SEADL,  SDS, PMS, CMS. | 45 minutes of interactive dialogue group lecture and followed by individualized and tailored rehabilitation 24 half-hour sessions over eight weeks for the intervention group | Significant improvement observed following 8 weeks intervention for the health-related quality of life (HRQOL) (p<0.001).  PD persons and their caregivers in experimental group also reported their mood elevations following the program. |
| --- | --- | --- | --- | --- | --- |
| 10. | Lewis et al. 2009 | To evaluate the effect of the Stress- Busting Program (SBP) for family caregivers. | Design: RCT  Sample: A total of 209 caregivers of ADRD patients  Outcome Measure: QoL for caregivers was determined using a variety of questionnaires that assess mental, social, and emotional health. | Phase 1, 2, and 3 of the Stress Busting Program:  Phase 1 of SBP: Simple relaxation strategies.  Phase 2 of SBP involved multicomponent program for caregivers of patients with ADRD. Caregivers are given a handbook, a relaxation/meditation CD, and a resource book for caregivers.  Phase 3: This program is adapted to PD caregivers | Caregivers’ experiences significant decrease in perceived stress, depression, burden. They also reported improved general health, social function, and mental health scores. |

Note: Parkinson's disease Questionnaire (PDQ-8), EQ-5D (EuroQoL-5 Dimensions), The Health Education Impact Questionnaire (Hei Q), Life satisfaction: LiSat-11 (Life Satisfaction Questionnaire-11), Fatigue: PFS-16 (Parkinson Fatigue Scale), Caregiver burden: ZBI-22 (Zarith Burden Interview-22), Curriculum evaluation: Chronic Disease Self-Management Program (CDSMP), Center for Epidemiologic Studies Depression Scale (CES-D), The Modified Caregiver Strain Index (MCSI), Visual Analog Scale (VAS), Parkinson's disease Questionnaire -39 (PDQ-39), The Scale of Quality of Life of Caregivers (SQLC), The Psychosocial Adjustment to Illness Scale (PAIS-SR), Patient Education Program Parkinson (PEPP), Self- rating Depression Scale (SDS), Belastungsfragebogen Parkinson Kurzversion (BELA-P-k), Belastungsfragebogen Parkinson Angehörige Kurzversion (BELA-A-k), Unified Parkinson Disease Rating Scale (UPDRS), Schwab and England Activities of Daily Living (SEADL), Global patient’s mood status (PMS), Caregiver’s mood status (CMS), Alzheimer's Disease Related Dementias (ADRD).

## RESEARCH QUESTIONS

1. What are the caregiver’s perception and needs on current education and training in the development of family caregiver-training program for managing persons with PD?
2. What is the level of physical functioning and QoL among persons with PD in Malaysia?
3. How valid and reliable is the family caregiver-training program for persons with PD?
4. Is the family caregiver-training module feasible and well accepted by the people with PD and their family caregivers?
5. What is the difference between pre and post score of functions, mobility and QoL of persons with PD who receive a structured, multi-component family caregiver-training program when compared to those receiving the usual intervention?
6. What is the difference between pre and post score of knowledge, burden and QoL of family caregivers who receive a structured, multi-component family caregiver-training program when compared to those receiving the usual intervention?
7. How cost effective is the family caregiver-training program for persons with PD and their family caregiver?
8. How sustainable is the family caregiver-training program for persons with PD in terms of retention of effect, compliance and number of dropouts?

## OBJECTIVES

- 1. **General Objectives**

To develop and validate a structured multi-component training module plus establish its feasibility, acceptance, clinical effects, cost effectiveness and sustainability for family caregivers of persons with PD.

## Specific Objectives

1. To explore the caregiver perception and needs related to education and training in the caregiving of persons with PD.
2. To determine the level of physical functioning and QoL among persons with PD in Malaysia.
3. To design and validate a structured multi-component family-training program and determine its reliability.
4. To develop the structured multi-component family-training program and determine its feasibility and acceptance.
5. To compare the pre and post score of functions, mobility and QoL between persons with PD receiving the structured multi-component family-training program and usual intervention.
6. To compare the pre and post score knowledge, QoL and burden between family members of persons with PD receiving the structured multi-component family- training program and a usual intervention.
7. To evaluate the cost effectiveness of the structured multi-component family-training program and control group.
8. To determine the sustainability of the structured multi-component family-training program among the family of persons with PD.

## HYPOTHESIS

- 1. **Null Hypothesis**

The structured multi-component family-training program is not more effective clinically and by cost, when compared with a usual intervention in improving functions, functional mobility and QoL of persons with PD, carer knowledge, carer QoL and burden among family members of PD when compared to the usual intervention. The family-training program is not sustainable and not well accepted by family and people with PD.

## Alternate Hypothesis

The structured multi-component family-training program is more clinical and cost-effective than usual intervention in improving functional mobility and QoL of persons with PD, carer knowledge, carer QoL and burden among family members of PD when compared to the usual intervention. The family-training program is sustainable and well accepted by family and people with PD.

## METHODOLOGY

- 1. **Overall Design, Time Scale and Venue**

This study utilizes instructional design based on quantitative and qualitative research approaches. ADDIE model will be used in developing the family caregiver-training program,

which involves the process of analysis, design, development, implementation and evaluation (ADDIE) of the program. This study consists of Phase I, II and III corresponding to the all five phases of ADDIE model as shown in Figure 1. This study encompasses needs analysis, FGD, pilot and a full scale single blinded randomized controlled trial (RCT) with an addition of cost- effectiveness analysis. This study will be conducted at the Universiti Kebangsaan Malaysia Medical Centre (UKMMC) in collaboration with the Malaysian Parkinson’s Disease Association (MPDA). The study duration will be from April 2022 to October 2024.

1.
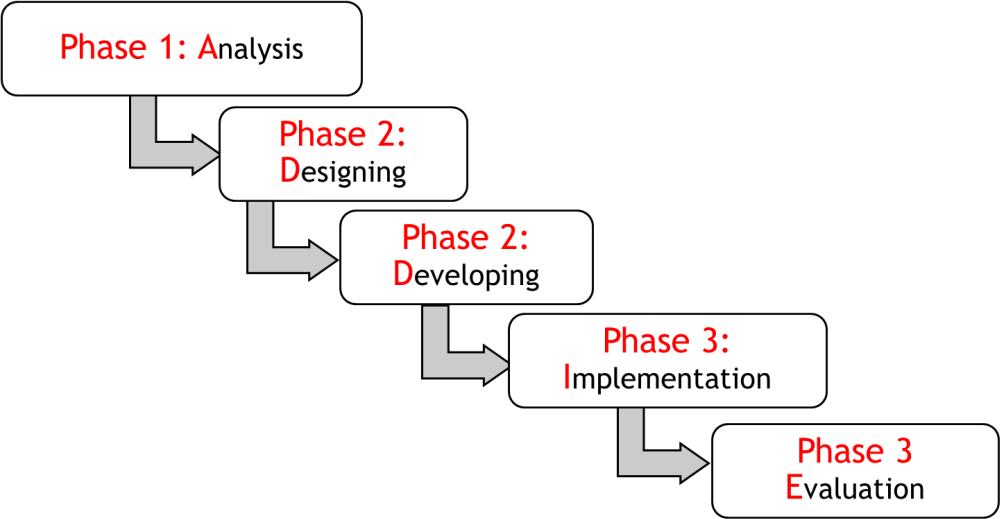
Literature Review
2. FGD
3. Analysis of function and QoL
   1. Early draft of Family Caregiver- Training Program
      1. Module Development
      2. Content Validation
      3. Pilot RCT
         1. Assessor blinded RCT

Figure 1 Overall design of the study based on ADDIE model

## PHASE I

Phase I of the study is the analysis phase of ADDIE model. Phase 1 of the study comprises of literature review, needs analysis of physical functioning and QoL of persons with PD and a FGD to aid in the design and development of family caregiver-training module. Generally, according to Muruganantham, (2015), analysis phase of ADDIE model comprise of needs assessment of the family caregiver-training program, problem identification of PD persons and their caregiver which is crucial to be addressed in the module plus goals and task analysis which includes determining solutions.

## Needs analysis of physical functioning and QoL of persons with PD

1. **Study design**

Needs analysis of physical functioning and QoL of persons with PD will utilize quantitative approach using cross sectional study design.

## Target population

Persons with PD in stage I and II.

## Sample population

Persons with PD in stage I and II, registered with the MPDA or referred to the Medical Rehabilitation Services Department of UKMMC.

## Inclusion and exclusion criteria of participants

Table 2 Eligibility criteria of participants for needs analysis

| **Inclusion criteria** | **Exclusion criteria** |
| --- | --- |
| 1. 50-75 years old 2. Stage I and II of Parkinson’s disease based on HY scale 3. Availability of family caregivers. | 1. Impaired physical function due to other conditions such as a recent fracture within the past 6 months, severe arthritis or other neurological disease such as peripheral neuropathy. 2. Have medical illness limiting participation in exercise, such as unstable angina and   uncontrolled hypertension. |

Hoehn and Yahr scale used for screening the eligibility of participants (persons with PD) are attached in Appendix 2.

## Sampling design and sample size calculation

A total of 196 participants will be recruited in this needs analysis from medical rehabilitation department from UKMMC or MPDA. The formula used to estimate sample size for this needs analysis will be according to Krejcie & Morgan (1970), using table for determining sample size from a given population that was derived from the formula (Krejcie & Morgan 1970). This formula was intended as the estimating population proportions at a specific probability and level of accuracy (Johnson & Shoulders 2019).

The formula used and its calculation was:

s = X^2^NP(1 – P) ÷ d^2^(N – 1) + X^2^P(1 – P)

s = (3.841)(400)(0.50)(1-0.50) ÷ (0.05)^2^ (400-1) + (3.841)(0.50)(1-0.50)

s = 196 participants

where, s is required sample size, X^2^ is the table value of chi-square for 1 degree of freedom at the desired confidence level (3.841), N is the population size, P is the population proportion (assumed to be .50 since this would provide the maximum sample size) and d is the degree of accuracy expressed as a proportion (.05).

## Flow chart of needs analysis of physical functioning and QoL of PD persons

Ethical approval will be obtained from UKMMC and the contact information will be collected from medical rehabilitation of UKMMC and MPDA. Participants will be contacted via WhatsApp based on their contact information obtained from the registry either from UKMMC or MPDA. Ethical approval will be obtained from UKMMC and letter of collaboration from MPDA as attached in appendix 3. Patient information sheet (appendix 4) and informed consent sheet (appendix 5) will be distributed to all participants who fit the eligibility criteria. Once eligible participant provide the informed consent, questionnaire containing three sections as explained in outcome measurement section will be provided via WhatsApp. Data will be analysed descriptively using SPSS and reported as frequency, percentage, mean, standard deviation. Flow of the needs analysis is described in Figure 1.

Report writing

Data analysis

Data collection using part II of MDS-UPDRS and PDQ-39

Provide patient information sheet and informed consent

Contact and screen participants for eligibility

Obtaining ethical approval

Figure 2 Flow chart of needs analysis study

## Outcome measurement tools

Data will be collected using outcome measurement tool as attached in appendix 6. The questionnaire will be created using Google form and be circulated via online platform such as WhatsApp to be answered by the participants (person with Parkinson’s disease). The questionnaire consists of three sections, namely demographic data, Movement Disorder Society-Unified Parkinson’s Disease Rating Scale (MDS-UPDRS) Part II: Motor Aspects of Experiences of Daily Living (M-EDL) and 39-Item Parkinson’s disease Questionnaire (PDQ- 39). Demographic data section contains questions regarding age, gender, race, marital status, working status, duration of Parkinson’s disease, stage of Parkinson’s disease and care taker.

Part II in MDS-UPDRS assess the motor of experiences of daily living. It consists of 13 self- reported items which are speech, saliva and drooling, chewing and swallowing, eating tasks, dressing, hygiene, handwriting, doing hobbies and other activities, turning in bed, tremor, getting out of bed, a car, or a deep chair, walking and balance and freezing. All items are scored on a scale from 0: normal, 1: slight, 2: mild, 3: moderate and 4: severe. MDS-UPDRS has high internal consistency with Cronbach’s Alpha 0.79 – 0.93 (Goetz et al. 2008). It shows comparative fit index > 0.90 for each part thus, the sum score for each part can be reported separately (Goetz et al. 2008).

The PDQ-39 assess quality of life. It consists of 8 dimensions which are mobility (10 items), activities of daily living (6 items), emotional well-being (6 items), stigma (4 items), social support (3 items), cognitions (4 items), communication (3 items) and bodily discomfort (3 items). Rating are made based on Likert scale from never, occasionally, sometimes, often and always. PDQ-39 shows acceptable internal consistency with Cronbach’s Alpha 0.59 –

0.94 and test-retest reliability with intra-class correlation coefficient 0.67 – 0.87 (Tan et al. 2004).

## (8) Statistical tool

Statistical Analysis Statistical Package for Social Sciences (SPSS) will be used and variables namely the level of physical functioning and QoL will be analyzed descriptively, presenting in mean and standard deviation.

## FGD

1. **Study design**

FGD utilizes qualitative approach using semi structured interview in FGD. Main feature of FGD in phase I, analysis is the interactive discussion of module development by a group of all participants (family caregivers) and a team of facilitators in a setting. This is the most popular and traditional focus group discussion format (O. Nyumba et al. 2018). Findings from the FGD along with literature review findings will be used in finalizing the training program content prior to its development using the ADDIE model.

## Target population

Family-caregiver of persons with PD in stage I and II.

## Sample population

Family-caregiver of persons with PD in stage I and II, registered with the MPDA or referred to the Medical Rehabilitation Services Department of UKMMC.

## Inclusion and exclusion criteria of participants

Table 2 Eligibility criteria of participants for FGD

| **Inclusion criteria** | **Exclusion criteria** |
| --- | --- |
| Main family caregiver who lives with the PD patient in stage I-II Hoehn and Yahr (HY) scale | 1. Providing part-time caregiving due to availability of maid/helper |
|  | 2. Presence of clinically diagnosed depression with a cutoff point of  ≥16 in Center for Epidemiologic  Studies Depression Scale (CES-D). |
|  | 3. Presence of mild cognitive impairment or dementia with a cutoff point of ≤26 in Montreal Cognitive  Assessment (MoCA). |

All instruments used for screening the eligibility of participants (family caregivers) are attached in Appendix 2.

## Sample size and number of FGD

Two groups of FGD consisting of a maximum of 12 participants (family caregivers) each will be conducted to gather their needs related to training and ideas and opinions in designing and developing a relevant structured, multi-component family caregiver-training program for people with PD. There will be a minimum of 2 session of FGD, until data saturation is obtained (O. Nyumba et al. 2018).

## Duration, Mode and Venue of FGD

Duration of FGD will be between one to two hours (O. Nyumba et al. 2018). The mode of session; either virtually of face to face will be determined by the preference of the participants. Virtual session will be held using Microsoft Teams and the session will be recorded. If participants agree for face to face session, FGD will be held in the physiotherapy department of UKMMC.

## Flowchart of FGD

Figure 3 illustrates the flow of FGD. Participants will be recruited via purposive sampling from Medical Rehabilitation Services Department of UKMMC and from MPDA based on the eligibility criteria as mentioned earlier. A minimum of two sessions of FGD will be conducted to discuss and its procedure will be briefed verbally and using a written information sheet as in Appendix 7. Participants will be asked to sign an informed consent form prior to the FGD as attached in Appendix 5. Arrangements on date and time for FGD will be based on the convenience of all participants. A quiet location will be determined at the Physiotherapy Unit at UKMMC if face to face session is preferred for the FGD or alternatively virtual session using Microsoft Teams will be arranged. The group will be asked to discuss issues pertaining the needs and care of PD patients according to a list of pre-prepared and piloted open-ended questions, then to suggest the content of the family training program. The researchers will play the role of a moderator and guide the focus group members on the discussion throughout the session. Views from each group member will be tape-recorded (face to face session) or recorded if it is a virtual session by another researcher who is also responsible to take field notes. To prevent fatigue, the maximum duration of each FGD will be limited to 2 hours and more sessions will be decided based on data saturation (O. Nyumba et al. 2018).

Adopt and pilot test to validate the questions developed for FGD

Screening participants for eligibility

Providing information sheet and obtaining informed consent

Participants gathered either virtually or face to face session

First FGD session: Introduction of the session by the facilitator

Moderator A begins semi strutured interview

Moderator B will be taking field notes

Transcription of data

2nd session of FGD as similar process as before

Decide data saturation or conduct more session of FGD

Thematic analysis

Report writing and using the data for Phase II

Figure 3 Flow chart of FGD

## Statistical analysis

Data analysis will be done according to the principles of qualitative research. All recorded data will be transcribed verbatim and thematic analysis will be performed. Triangulations in data analysis will be ensured by including an independent researcher and respondents’ validation will be done prior to finalizing the FGD findings. The steps of performing thematic analysis is as described below (Maguire & Delahunt, 2017):

- 1. Data familiarization
     1. Data transcriptions and multiple reading (read and re-read) of the transcripts
     2. Writing early impression upon reading
  2. Generation of initial code
     1. Meaningful and systematic data organization to highlight important pattern
     2. Inductive analysis will be used for initial coding
     3. Step 1 of inductive analysis involves two types of approaches in vivo coding and structural coding. Participants’ own word will be used to generate code in in vivo coding and in structural coding, sections of the data will be coded to address the questions from the open – ended survey questions.
     4. Step 2 involves line by line coding to add on more details to the initial coding.
  3. Theme searching
     1. Preliminary primary themes and subthemes will be sorted from relevant coded data extracts
  4. Theme reviewing
     1. Review each data set mapped to the appropriate theme
     2. May involve appraisal, modification and development of the themes
  5. Theme defining
     1. Themes will be finalized
  6. Report writing

## PHASE II

Phase II of the study involves the designing and development phase of ADDIE model. The design phase involves planning on strategy to achieve goal set in analysis stage and resource identification to develop the module (Muruganantham, 2015). While, the development phase begins with construction and development of the module and its instrument plus instructions using media software and documentation (Muruganantham, 2015).

## Module designing

Two researchers will be involved in the early draft of module and the storyboard based on the phase I FGD report and literature review. Researchers will also meet a group of subject experts for discussion on their respective components for their module. Team of experts in this study consist of neurologist, nurses, physiotherapist (PT), occupational therapist (OT), psychologist, speech and language pathologist (SLP), and dietitians. Module will be written in both English and Bahasa Malaysia. The module design phase consist of:

- - - 1. Determining the study objective
      2. Overall module content
      3. Activities of each component
         1. Medical and nursing management
         2. Rehabilitation management – PT, OT, SLP, psychologist and dietitians
      4. Detailed description and instruction of each activity
      5. Flow of the module contents

## Instrument designing

Variables identified for this study involves functions, mobility and QoL of persons with PD while for caregivers involves knowledge of PD, burden of caregiving and QoL. There are four instruments which does not require validation as its psychometric properties are already established, namely:

- - - 1. Movement Disorder Society-Unified Parkinson’s Disease rating scale (MDS-UPDRS) and Timed Up and Go (TUG) test which measures functionality and mobility of persons with PD respectively.
      2. EuroQoL-5 Dimensions (EQ-5D) and Malay version of Zarith Burden Interview (ZBI) measuring the caregiver’s QoL evaluating and burden of caregiving respectively.

However, translation and validation is compulsory for questionnaire measuring knowledge of PD among family caregivers using questionnaire developed by Moore and Knowles, (2006). A copy of approval email to use the questionnaire of Moore and Knowles (2006) is attached in appendix 8. The steps involved for the translation of the knowledge of PD among caregivers instrument is shown in Figure 4 based on a study by Sousa and Rojjanasrirat (2011). While validation of Bahasa Malaysia version of EQ-5D among persons with PD in Malaysia will be conducted by performing full psychometric testing. Language and subject experts in the instrument design phase will be recruited via purposive sampling from UKM. Approval is also obtained to use the Malay version of ZBI as attached in appendix 8.

Identify one language and subject expert (n=2) for English to Bahasa Malaysia translation

Comparison of the two translated versions of the instrument

Identify one language and subject expert (n=2) for Bahasa Malaysia to English translation

Blind back-translation of the preliminary initial translated version of the instrument

Comparison of the two back-translated versions of the instrument

Pilot testing of the pre-final version of the instrument in the target language

Preliminary psychometric testing of the pre-final version of the translated instrument

Full psychometric testing of the pre-final version of the translated instrument

in a sample of the target population, caregivers of persons with PD

Figure 4 Flow chart of knowledge of PD among caregivers instrument design

## Module development

Procedure 1: Designed module will be discussed with the team of experts mentioned earlier to approve for content and storyboard to commence development in dual language. Pre- developed module will be submitted to the illustrator for better quality development.

Procedure 2: Validation of the developed module through percentage calculation method (PCM) and content validity index (CVI)

- - - 1. Recruitment of experts meeting the eligibility criteria as below via purposive sampling from UKMMC and UKM:
         1. Inclusion criteria: Minimum Masters or PhD in neurology or geriatric care with at least three years of clinical experience managing PD persons and caregivers.
         2. Exclusion criteria: Experts who are not able to provide informed consent.
      2. A total of 7 experts namely neurologist, nurses, OT, PT, SLP, psychologist and dietitians will be recruited based on the eligibility criteria to answer Module Validity Testing Questionnaire (Russell, 1974). The questionnaire is based on 5 point Likert scale with score 1 (highly agree) and score 5 (totally disagree). Five items will be analyzed;
         1. Suitability of module content to family caregiver and persons with PD
         2. Successful implementation of the module content
         3. Module content is suitable with allocated time
         4. Module content may provide positive impact
         5. Module content may provide desired results among family caregiver and persons with PD
      3. Percentage calculation method to determine the level of module content validity. This will be obtained by dividing the number of scores filled by the experts to the actual score and multiplied by 100. Acceptable value will be 70% and above (Tuckman and Waheed, 1981 & Sidek and Jamaluddin, 2005).
      4. Content validity index will be calculated as follows;
         1. Divide the 5 point ordinal Likert scale into two categories; score 0, 1 and 2 (agreed) = give score 0 and above score 2 is disagreed = give score 1.
         2. Compute each items of the five criteria from based on the two categories by each experts
         3. CVI of each items are calculated by dividing the sum of the evaluators agreed with sum of evaluator as shown in example of the table below.

Procedure 3: Reliability analysis. Analysis of Cronbach’s alpha coefficient will be calculated. (Sidek & Jamaluddin, 2005).

## Formative evaluation: Pilot RCT

1. **Study design**

Quantitative approach using experimental design, a pilot RCT.

## Target Population

The target population in this study are PD patients of stage I and II, and their family caregivers.

## Eligibility criteria Inclusion criteria:

- 1. PD patients

1. 50-75 years old
2. Stage I and II of Parkinson’s disease based on HY scale
3. Availability of family caregivers.
   1. Family caregivers
      1. Main caregiver who lives with the PD patient

## Exclusion criteria:

1. PD patients
2. Impaired physical function due to other conditions such as a recent fracture within the past 6 months, severe arthritis or other neurological disease such as peripheral neuropathy.
3. Have medical illness limiting participation in exercise, such as unstable angina and uncontrolled hypertension.
4. Family caregiver:
   1. Providing part-time caregiving due to availability of maid/helper
   2. Presence of clinically diagnosed depression with a cutoff point of ≥16 in CES-D.
   3. Presence of mild cognitive impairment or dementia with a cutoff point of ≤26 in MoCA.

## Sampling Frame

Main sampling frame for this study phase is all persons with PD registered with the MPDA or PD patients referred to the Medical Rehabilitation Services Department of UKMMC since January 2021 and their main family-caregiver. MPDA registry and patient’s registry maintained by the neurology rehabilitation unit of the department will be accessed to identify PD patients.

## Sampling Method and Sample Size Estimation

Eligible PD patients will be recruited using a purposive sampling method. A group of 10 pairs will be recruited for this study (10 caregivers and 10 patients per group) based on the latest recommendation by Whitehead et al. (2016) for a minimum of 10 participants per trial arm for pilot study.

## Study Procedure

1. Explanation about the study will be provided using information sheet as attached in appendix 9 and eligible PD patients and family-caregivers will be requested to sign an informed consent form prior to participation in the study.
2. Data on social-demography will be compiled and baseline measurements of functionality, mobility and QoL of persons with PD, caregiver knowledge, burden and QoL will be conducted and analyzed to ensure the two groups are comparable at baseline.
3. The participants will then be randomly allocated into either the experimental group or the control group using ‘sealed opaque envelope’ technique by health personnel blinded to the study.
4. Training sessions will be conducted by a group of multidisciplinary health care team at the Physiotherapy Unit of UKMMC and family caregivers will be required to demonstrate the components. Training module booklet containing a multi-component training materials will be provided to facilitate care at home and the content of the module consisting of multi-care physical and psychosocial tasks and guidance on health care for PD patients will be established based on findings of FGD.
5. The experimental group will receive a structured, comprehensive family training programme while the control group will receive usual intervention which normally consist of advice from treating medical officers and usual therapy program by rehabilitation professionals as necessary. The usual program consists of physiotherapy (functional training, strength and balance training) and occupational therapy (activities of daily living training and self- care).
6. Both groups will receive the interventions on a weekly basis for 12 weeks and followed-up (telephone) weekly to ensure compliance and monitor any arising problems or adverse effects.
7. Measurement of outcomes will be done at week 0 and 12 of the intervention, while program feasibility and acceptance will be assessed at week 12 as depicted in Figure 5.

Will be excluded based on

exclusion criteria

Persons with PD and their family caregivers will be screened in medical rehabilitation department of UKMMC (n=40)

Eligible participants will be randomized based on simple random sampling (n=40) and baseline measurement will be taken


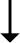

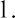

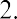

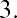

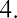

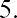


Assessment of outcomes after 12th week

Functions and mobility measured using TUG and MDS-UPDRS respectively

QoL of patient and family caregivers measured using EQ-5D.

Caregiving burden measured using Zarith Burden

Interview.

Knowledge of carer measured using adapted , translated and validated questionnaire Feasibility and acceptance assessment

Assessment after 12th week

(n=40), intention to treat approach)

Allocated to control group; usual intervention (n=20)

Allocated to experimental group; family caregiver training module (n=20)

Training will be given to caregivers before intervention

Results compilation and reporting

Figure 5 Flow Chart of Phase II

## Outcome measurement tools for pilot RCT

All outcome assessment will be conducted by a blinded assessor at week 0 (pre intervention) and week 12. Feasibility and acceptance assessment will be evaluated based on the clinical effects, rate of adherence, drop-outs and feedback from the program participants only after week 12. Clinical effect outcomes measured on persons with PD and family caregivers are summarized in Table 3. Description and psychometric properties of the outcome measures are depicted in detailed in summative evaluation. Sample of outcome measurement tool is attached in the Appendix 10.

Table 3 Outcome measures used for family caregivers and persons with PD

| **Persons with PD variables** | **Outcome measurements tools** |
| --- | --- |
| I. Mobility | Timed Up and Go (TUG) test |
| II. Motor and non-motor functions | The Movement Disorder Society-Sponsored Revision of the Unified Parkinson's Disease  Rating Scale (MDS-UPDRS) |
| III. QoL | EQ-5D |
| **Family Caregivers of Persons with PD**  **variable** | **Outcome measurements** |
| I. Knowledge level of carer | Adapted and translated from study by  Moore and Knowles, 2006 |
| II. Caregiving burden | Zarith Burden Interview |
| III. QoL | EQ-5D |

## Statistical tools for pilot RCT

Formative evaluation of pilot RCT will adhere to the same statistical tool depicted in section

7.4.8 of statistical tools in summative evaluation. Summary of the statistical tests used as per objective is highlighted in Table 4.

Table 4 Statistical tests mapped to objectives for phase II

| **Objectives** | **Statistical test** |
| --- | --- |
| To develop the structured multi-component family-training  program and determine its feasibility and acceptance |  |
| Clinical effects | 6x2 Mixed Model ANOVA |
| Number of drop outs Adherence Feedback  Adverse events | Descriptive statistics (Mean, standard deviation and percentage) |

## PHASE III – Implementation and summative evaluation

Phase III involves implementation of the module through an assessor blinded RCT and a summative evaluation.

## Study design

A single-blind RCT followed by a costing analysis.

## Target Population

The target population in this study is PD patients of stage I and II, and their family caregiver.

## Eligibility Criteria INCLUSION CRITERIA:

1. PD patients
   1. Age 50-75 years old
   2. Stage I and II of Parkinson’s disease based on HY scale
   3. Availability of family caregivers.
2. Family caregivers
   1. Main caregiver who lives with the PD patient

## EXCLUSION CRITERIA:

1. PD patients
   1. Impaired physical function due to other conditions such as a recent fracture within the past 6 months, severe arthritis or other neurological disease such as peripheral neuropathy.
   2. Have medical illness limiting participation in exercise, such as unstable angina and uncontrolled hypertension.
2. Family caregiver:
   1. Providing part-time caregiving due to availability of maid/helper
   2. Presence of clinically diagnosed depression with a cutoff point of ≥16 in CES-D.
   3. Presence of mild cognitive impairment or dementia with a cutoff point of ≤26 in MoCA.

## Sampling Frame

Main sampling frame for this study phase is all persons with PD registered with the MPDA or persons with PD referred to the Medical Rehabilitation Services Department of UKMMC since January 2021 and their main family caregiver. MPDA registry and patient’s registry maintained by the neurology rehabilitation unit of the department will be accessed to identify persons with PD.

## Sampling Method and Sample Size Estimation

Eligible persons with PD will be recruited using a non-probability purposive sampling method. Selected participants (persons with PD) will then be allocated into either the test group (family-training program) or the control group (usual intervention) using simple randomization method. Sample size is calculated using Gpower software version 3.1. This study will use 6x2 Mixed Model Anova with post hoc analysis using Bonferroni. Therefore F-test (ANOVA repeated measures, within-between interactions) is chosen in Gpower software. Study power is set at 95%, alpha set at 0.05 and effect size of 0.41 was chosen based on the effect sizes for the primary outcomes of clinical interest from the study by Bueno et al. (2017). Based on sample size calculation, a total of 18 participants are required. Hence a minimum of nine PD patients and nine family caregivers (n=18 per group) will be recruited in each group which makes it a total of 36 participants in this study. Dropouts are not considered because intention-to-treat analysis will be used and all participants who are enrolled at baseline will be included in the final analysis.

## Study Procedure

**(A) Recruitment and randomization**

Explanation about the study will be provided to participants as attached in the information sheet of appendix 11 and eligible persons with PD and family caregivers will be requested to sign an informed consent prior to participation in the study. Data on social-demography will be compiled and baseline measurements of functionality mobility and QOL of PD patients, caregiver knowledge, burden and QoL will be conducted and analyzed to ensure the two groups are comparable at baseline. The participants will then be randomly allocated into either the experimental group or the control group using ‘sealed opaque envelope’ technique by health personnel blinded to the study.

## (B) Family caregiver training module and usual intervention

Table 5 highlights the content of experimental and control group training. The experimental group will receive a structured, comprehensive family caregiver-training program while the control group will receive usual intervention which normally consist of advice from treating medical officers and usual therapy program by rehabilitation professionals as necessary. The usual program consists of physiotherapy (functional training, strength and balance training) and occupational therapy (activities of daily living training and self-care). Both groups will receive the interventions on a weekly basis for 12 weeks and followed-up weekly to ensure compliance and monitor any arising problems or adverse effects.

Table 5 Intervention summary in experimental and usual intervention

| Characteristics | Family caregiver training module | Usual intervention |
| --- | --- | --- |
| Total duration | 60-90 minutes for overall module | 60-90 minutes |
| Delivered by | Family caregiver | Healthcare professionals |
| Content | Multidisciplinary approach; various perspective from neurologist, nurses, PT, OT, SLP, psychologist, dietitians. | Individualized and as referred by the neurologist based on problems arised |
| Location | Home based | Hospital based |
| Frequency | As needed for 12 weeks | Appointment based for 12 weeks |

## (C) Training for family caregiver in experimental group

Training module booklet containing a multi-component training materials will be provided to facilitate care at home and the content of the module consisting of multi-care physical and psychosocial tasks and guidance on health care for PD. Training sessions will be conducted by a group of multidisciplinary health care teams at the Physiotherapy Unit of UKMMC. Thereafter, family caregivers will be asked to demonstrate the components to proceed with the module at home. Experts from each component will decide if retraining session is necessary.

## (C) Measurement of outcomes

Outcomes will be evaluated at week 0, 12 and at 6-month follow up. Program sustainability will be assessed at a 6-month follow up by a blinded assessor**.** Summary of the study procedure is as attached in the Figure 6.

Will be excluded based on

exclusion criteria

Persons with PD and their family caregivers will be screened in medical rehabilitation department of UKMMC (n=36)

Eligible participants will be randomized based on simple random sampling (n=36) and baseline measurements will be taken

Assessment after 12th week (n=36, intention to treat approach)

Allocated to control group; usual intervention (n=18)

Allocated to experimental group; family caregiver- training program (n=18)


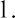

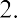

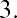

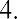


Results compilation and reporting

Sustainability assessment of outcomes after 6 months

1. Sustainability of effects based on clinical effects measured at week 12
2. Adherence, compliance, drop outs and adverse events.

Assessment of outcomes after 12th week

Functions and mobility measured using MDS-UPDRS and TUG respectively

QoL of patient and family caregivers measured using EQ-5D.

Caregiving burden measured using Zarith Burden Interview.

Knowledge of carer measured using adapted , translated and validated questionnaire

Figure 6 Flow Chart of Phase III

## Summative evaluation

All outcome assessment will be conducted by a blinded assessor at week 0, week 12 and at a 6-month follow up.

## ASSESSMENT OF THE CLINICAL EFFECTS OF THE MODULE USING OUTCOME MEASUREMENT TOOLS

Summative evaluation measures the clinical effect of the module as tabulated in Table 3 which summarizes the outcome measurement for each variable used for persons with PD and their family caregivers.

- - - 1. Mobility is measured using TUG. A chair will be placed on one end of the 3 meters (m) and a cone on the other end of 3m. Participants will be asked to stand and walk for 3m, turn around the cone, walk again and sit down. Stopwatch will be started as participants rises from the chair and will be stopped as participants sit down. TUG has excellent test- retest reliability among PD population with intraclass correlation coefficient (ICC) ranging from 0.80 (Huang et al. 2011) to 0.85 (Steffen & Senney, 2008). A study by Bennie et al. (2003) reported TUG has excellent inter rater and intra rater reliability of ICC of 0.99 and

0.98 respectively. A study by Foreman et al. (2011) reported that TUG has good construct validity for PD, area under the curve value ranging from 0.68 during “on” stage and 0.80 during “off” stage when compared to the contrast test, Functional Gait Assessment. The authors further reported TUG is responsive during both “on” and “off” period of PD. Scoring of TUG test is the time taken in seconds, (s) to complete one lap as instructed. Brusse et al. (2005) suggested the mean normative TUG test data for PD persons is

14.8s with a minimum of 4.85 seconds for minimal detectable change (Dal Bello-Haas et al. 2011).

- - - 1. Functions namely motor and non-motor functions will be evaluated using therapist rated MDS-UPDRS. MDS-UPDRS is a revision of UPDRS and has total four parts which will be administered by the therapist. Part I and II consist of 13 questions each evaluating non motor and motor experiences of daily living respectively. Part III consist of 18 questions of motor examination while part IV asks six questions on motor complications. All parts of MDS-UPDRS has 5 response option, ranging from 0 (normal) to 4 (severe).The higher total summed score indicates greater functional decline among PD persons. It takes only 30 minutes to administer the questionnaire (Shirley Ryan Ability Lab, 2022). MDS-UPDRS shows excellent reliability and concurrent validity as comparative fit index shows greater than or equal to 0.90 and high correlations (r=0.96) between the MDS-UPDRS and the original UPDRS Part III (Pal & Goetz, 2013).
      2. QoL of family caregivers and persons with PD will be assessed using EQ-5D, a standardized, non-disease-specific tool to measure health states (EuroQoL 1990). This questionnaire consists of 2 parts; the EQ-5D descriptive system and the EQ visual analogue scale (EQ VAS) (EuroQoL 2015). In this study, EQ- 5D-5L descriptive system with five levels of response for each dimensions will be used. The EQ-5D-5L consists of five questions and each question has a single digit response option and participants can only select any one option per question. Each question resembles one item of QoL namely; mobility, self-care, usual activities, pain/discomfort, and anxiety/depression. The selected digits for all five questions will combined into a five-digit number describing the participant’s health state. The five digit number of each EQ-5D-5L health states will then be entered into the EQ-5D-5L index value calculator to generate single index value known as health utility value based on specific value sets of respective country (Van Reenen & Janssen 2015). The utility score ranging between ‘0’ and ‘1’ represent death and perfect health respectively (Szende, Oppe & Devlin 2007). To date, utility value set for Asian country was only available for Japan and Thailand (EuroQol 2016; Szende, Oppe & Devlin 2007), hence Thailand value set of EQ-5D-5L will be used in this study (Nor Azlin, 2013).

The second part of EQ-5D is a 20cm scale called EQ VAS, to assess the patient’s perceived health status. ‘X’ mark will be placed on the EQ VAS scale which has two endpoints labelled “worst health” and “best health”. The marked value will then be written in the available column of the scale (Van Reenen & Janssen 2015). English and Malay version of EQ-5D-5L is available to be used. EQ-5D has good convergent validity as there is strong associations between the EQ-5D and PDQ-39 or SF-36 subscores of both outcome measures for persons with PD (Schrag, 2000). Translated version of EQ-5D has satisfactory predictive validity and test retest reliability among generic Malaysian population with ICC ranging <0.01 to 0.92 and the spearman rank correlation coefficient value ranging from 0.61 to 0.86 (Varatharajan & Chen, 2012). Even though, a study by Luo and colleagues (2009) conducted in Singapore proved that EQ-5D is valid in PD person, this study will establish the validity and reliability of this instrument among Malaysian PD persons as highlighted in instrument design.

- - - 1. Burden of caregivers will be measured using ZBI, the Malay version. Family caregivers will be asked to specify the extent of burden during caregiving in the 22 items questionnaire. Scores range from never (score 0) to nearly always (score 4). Total scores will be obtained from summing the total score of all 22 items with a maximum score of

88. Greater the score indicate greater the burden and a score above 17 indicate high burden (Bedard et al, 2001). Reliability was good for all scales (ordinal alpha 0.89-0.95). A recent study by Shim, Ng and Drahman (2017) reported high internal consistency of

0.898 and area under the curve of 0.786 which indicates excellent reliability and validity respectively of Malay version of ZBI among Malaysian population.

- - - 1. Knowledge of caregiver questionnaire will be adapted, translated into Bahasa Malaysia and validated based on the study by Moore and Knowles, (2006). The questionnaire is expected to have 13 items with scoring of true or false for each item. All 13 items will be summed to produce a total score with higher score indicating higher level of knowledge.

## COST-EFFECTIVENESS EVALUATION

To measure cost-effectiveness of the program, costing analysis will be performed from the societal perspective (both patients/caregivers' and health care providers' perspectives) using an activity-based costing approach. Data on direct costs (e.g. cost of equipment/medication, human resource, transportation, etc) and indirect costs (e.g. loss of income/productivity) of the two interventions will be gathered through interviews with the caregivers and health care providers using a standard costing proforma. Cost-effectiveness of the intervention will then be determined by looking at cost per Quality adjusted life years (QALY) and based on average cost- effectiveness ratio (ACER) and incremental cost-effectiveness ratio (ICER). The Malaysian gross domestic produce (GDP) for the year 2022 will be used in the interpretation of the ACER and ICER data.

## ASSESSMENT OF PROGRAM SUSTAINABILITY

Program sustainability will be assessed by measuring all participants’ (persons with PD and their family caregiver) outcomes again at a 6-month follow-up and comparing the participants' variables with previously measured data. The aim is to determine if the positive effects of the training program can be maintained at least for 6 months of the program completion. Sustainability also will be measured based on compliance to exercise, adherence to the module component and dropout rate.

## Statistical Tools

Statistical Analysis Statistical Package for Social Sciences (SPSS) will be used and variables will be analyzed descriptively, presenting mean and standard deviation. Intention to treat analysis will be performed. Effects of the interventions and comparison between groups will be analyzed using 6 x 2 Mixed Model ANOVA. The level of significance is set at *p*<0.05. Mixed model ANOVA will provide results for main effect of time (before and after therapies), main effect of group (experimental and control group), time and group interaction effect. Statistically significant main effect of time highlights significant changes in the dependent variables following interventions. Statistically significant main effect of group highlights the significant difference between experimental and control groups on the outcomes. Statistically significant time and group interaction effect highlights if experimental group yield the desired effect on dependent

variable significantly compared to control group (Field 2009). Besides aforementioned effects, partial eta squared (ηp²) will also analyzed to display the degree of influence of the independent variable. Cohen (1988) stated that the effect is large for ηp²≥0.14, medium for 0.14>ηp²≥0.06 and small for ηp²≤0.01. Comparison of costs of the two interventions will be done using independent samples *t* test. Statistical tests mapped to objectives in phase III is presented in Table 6.

Table 6 Statistical tests mapped to objectives for phase III

| **Objectives** | **Statistical test** |
| --- | --- |
| To compare the pre and post score of functions, mobility and QOL between persons with PD receiving the structured multi-component family-training program and control group.  To compare the pre and post score knowledge, QOL and burden between family members of persons with PD receiving the structured multi-component family-training  program and control group. | 6x2 Mixed Model ANOVA |
| To evaluate the cost effectiveness of the structured multi-  component family-training program and control group. | Independent sample *t* test |
| To determine the sustainability of the structured multi- component family-training program among the family of  persons with PD. |  |
| - Retention of effect | 6x2 Mixed Model ANOVA |
| - Compliance - Adherence - Drop outs | Descriptive statistics (Mean, standard deviation and percentage) |

## Ethical Considerations

Approval to conduct the study in UKMMC will be obtained from the ethics committee of UKMMC before the commencement of the trial. The trial will be registered in the Australian and New Zealand clinical trial registry (ANZCTR) prior to commencement. All participants (persons with PD and their family caregivers) will be given an information sheet, and informed consent will be obtained before the intervention.

1. **Task Analysis** Attached in Appendix 12
2. **Gantt Chart** Attached in Appendix 13
3. **Budget** Attached in Appendix 14

## References

A'Campo, L. E., Wekking, E. M., Spliethoff-Kamminga, N. G., Le Cessie, S., & Roos, R. A. 2010. The benefits of a standardized patient education program for patients with Parkinson's disease and their caregivers. *Parkinsonism & related disorders*, *16*(2), 89–95. https://doi.org/10.1016/j.parkreldis.2009.07.009

A’campo, L. E. I., Spliethoff-Kamminga, N. G. A., Macht, M., & Roos, R. A. C. 2010. Caregiver education in Parkinson’s disease: formative evaluation of a standardized program in seven European countries. *Quality of Life Research*, *19*(1), 55-64.

Bagheri, S., Valizadeh Zare, N., Mazlom, S. R., Mohajer, S., & Soltani, M. 2019) Effect of Implementing Family-Centered Empowerment Model on Burden of Care in Caregivers of the Elderly with Parkinson's Disease. *Evidence Based Care*, *9*(3), 41-48.

Bédard, M., Molloy, D. W., Squire, L., Dubois, S., Lever, J. A., & O'Donnell, M. 2001. The Zarit Burden Interview: a new short version and screening version. *The gerontologist*, *41*(5), 652-657.

Bouça-Machado, R., Gonçalves, N., Lousada, I., Patriarca, M. A., Costa, P., Nunes, R., Dias, S., Caldas, A. C., Valadas, A., Lobo, P. P., Guedes, L. C., Rosa, M. M., Coelho, M., & Ferreira, J. J. 2020. Patients and Health Professional's Perspective of Functional Mobility in Parkinson's Disease. *Frontiers in neurology*, *11*, 575811.

<https://doi.org/10.3389/fneur.2020.575811>

Bennie, S., Bruner, K., Dizon, A., Fritz, H., Goodman, B., & Peterson, S. 2003. Measurements of balance: comparison of the Timed" Up and Go" test and Functional Reach test with the Berg Balance Scale. *Journal of Physical Therapy Science*, *15*(2), 93-97.

Brusse, K. J., Zimdars, S., Zalewski, K. R., & Steffen, T. M. 2005. Testing functional performance in people with Parkinson disease. *Physical therapy*, *85*(2), 134–141.

Bueno, M. E. B., Andrello, A. C. D. R., Terra, M. B., Santos, H. B. C. D., Marquioli, J. M., & Santos, S. M. S. 2017. Comparison of three physical therapy interventions with an emphasis on the gait of individuals with Parkinson’s disease. *Fisioterapia em Movimento*, *30*, 691-701.

Chaplin, H., Hazan, J., & Wilson, P. 2012. Self-management for people with long-term neurological conditions. *British journal of community nursing*, *17*(6), 250-257.

Cohen, J. 1988. Statistical Power Analysis for the Behavioural Sciences. Ed. Ke-2. New York: Lawrence Erlbaum Associates.

Dal Bello-Haas, V., Klassen, L., Sheppard, M. S., & Metcalfe, A. 2011. Psychometric Properties of Activity, Self-Efficacy, and Quality-of-Life Measures in Individuals with Parkinson Disease. *Physiotherapy Canada. Physiotherapie Canada*, *63*(1), 47–57. <https://doi.org/10.3138/ptc.2009-08>

EuroQoL. 2016. EQ-5D-5L Value sets. <http://www.euroqol.org/about-eq-5d/valuation-of-eq-> 5d/eq-5d-5l-value-sets.html.

Field, A. 2009. *Discovering Statistics using SPSS*. Ed Ke-3. Canada: SAGE publication.

Gultekin, M., Erturk, G., Ekinci, A. and Mirza, M. 2017. The Level of knowledge of Parkinson’s disease among non-professional caregivers in a movement disorders center in Turkey. Parkinson’s Disease, 3, 1–4.

Guo, L., Jiang, Y., Yatsuya, H., Yoshida, Y. and Sakamoto, J. 2009. Group education with personal rehabilitation for idiopathic Parkinson's disease, Canadian Journal of Neurological Sciences, 36 (1), 51-59

Hellqvist, C., Dizdar, N., Hagell, P., Berterö, C., & Sund‐Levander, M. 2018. Improving self‐ management for persons with Parkinson's disease through education focusing on management of daily life: Patients’ and relatives’ experience of the Swedish National Parkinson School. *Journal of Clinical Nursing*, *27*(19-20), 3719-3728.

Huang, S. L., Hsieh, C. L., Wu, R. M., Tai, C. H., Lin, C. H., & Lu, W. S. 2011. Minimal detectable change of the timed "up & go" test and the dynamic gait index in people with Parkinson disease. *Physical therapy*, *91*(1), 114–121. https://doi.org/10.2522/ptj.20090126

Foreman, K. B., Addison, O., Kim, H. S., & Dibble, L. E. 2011. Testing balance and fall risk in persons with Parkinson disease, an argument for ecologically valid testing. *Parkinsonism & related disorders*, *17*(3), 166–171. https://doi.org/10.1016/j.parkreldis.2010.12.007

Kessler, D., & Liddy, C. 2017. Self-management support programs for persons with Parkinson’s disease: An integrative review. *Patient education and counseling*, *100*(10), 1787-1795.

Harvanshah, M. 2019. Malaysian Parkinson's disease patients expected to rise five fold: here is what you need to know. Malay Mail. https://[www.malaymail.com/news/life/2019/04/11/malaysian-parkinsons-disease-](http://www.malaymail.com/news/life/2019/04/11/malaysian-parkinsons-disease-) patients-expected-t o-rise-fivefold-heres-what/1742188

Jenkinson, C., Dummett, S., Kelly, L., Peters, M., Dawson, J., Morley, D., & Fitzpatrick, R. 2012. The development and validation of a quality of life measure for the carers of people with Parkinson’s disease (the PDQ-Carer). *Parkinsonism & Related Disorders*, *18*(5), 483- 487.

Johnson, D. & Shoulders, C. 2019. Beyond Magic Words and Symbols: Rethinking Common Practices in Quantitative Research. Journal of Agricultural Education 60(3)

Kalra, L., Evans, A., Perez, I., Melbourn, A., Patel, A., Knapp, M., & Donaldson, N. 2004. Training carers of stroke patients: Randomised controlled trial. Bmj, 328(7448), 1099. https://doi.org/10.1136/bmj.328.7448.1099

Kessler, D., & Liddy, C. 2017. Self-management support programs for persons with Parkinson’s disease: An integrative review. *Patient education and counseling*, *100*(10), 1787-1795.

Kouli, A., Torsney, K.M., Kuan, W.L. 2018. Parkinson's disease: Pathogenesis and clinical aspect. e-book. Available at: <https://www.ncbi.nlm.nih.gov/books/NBK536722>. [Accessed date: 16 Feb 2022].

Krejcie, R. V. & Morgan, D.W. 1970. Determining Sample Size for Research Activities.

Educational and Psychological Measurement 30(3): 607–610

Krishnan Vasanthi, R., & Choo Kher Ying, A. . 2021. A Survey Of Perceived Barriers And Motivators Towards Exercise Among People With Parkinson’s Disease In Malaysia. *IIUM Medical Journal Malaysia*, *20*(3). https://doi.org/10.31436/imjm.v20i3.1645

Lewis, S. L., Miner-Williams, D., Novian, A., Escamilla, M. I., Blackwell, P. H., Kretzschmar, J. H., Arévalo-Flechas, L. C., & Bonner, P. N. 2009. A stress-busting program for family caregivers. *Rehabilitation nursing : the official journal of the Association of Rehabilitation Nurses*, *34*(4), 151–159. https://doi.org/10.1002/j.2048-7940.2009.tb00271.x

Lidstone, S. C., Bayley, M., & Lang, A. E. 2020. The evidence for multidisciplinary care in Parkinson's disease. *Expert review of neurotherapeutics*, *20*(6), 539–549. https://doi.org/10.1080/14737175.2020.1771184

Luo, N., Low, S., Lau, P. N., Au, W. L., & Tan, L. C. (2009). Is EQ-5D a valid quality of life instrument in patients with Parkinson's disease? A study in Singapore. *Interventions*, *2*, 3.

Lyons, K. S., Zajack, A., Greer, M., Chaimov, H., Dieckmann, N. F., & Carter, J. H. 2021. Benefits of a Self-Management Program for the Couple Living With Parkinson's Disease: A Pilot Study. *Journal of applied gerontology : the official journal of the Southern Gerontological Society*, *40*(8), 881–889. https://doi.org/10.1177/0733464820918136

Maguire, M., & Delahunt, B. 2017. Doing a thematic analysis: A practical, step-by-step guide for learning and teaching scholars. *All Ireland Journal of Higher Education*, *9*(3).

Maresove, P., Klimova, B. and Kuca, K. 2017. Medical and non-medical costs of Parkinson disease - comparison of Europe, USA, Asia and Australia. Ceska Slov Farm. Spring; 66(1), 3-8.

Martinez-Martin, P., Macaulay, D., Jalundhwala, Y. J., Mu, F., Ohashi, E., Marshall, T., & Sail,

K. 2019. The long-term direct and indirect economic burden among Parkinson's disease caregivers in the United States. *Movement disorders : official journal of the Movement Disorder Society*, *34*(2), 236–245. https://doi.org/10.1002/mds.27579

Moore, S., & Knowles, S. 2006. Beliefs and knowledge about Parkinson’s disease. *E-Journal of Applied Psychology: Clinical and Social Issues*, *2*(1), 15-21.

Mosley, P. E., Moodie, R., & Dissanayaka, N. 2017. Caregiver Burden in Parkinson Disease: A Critical Review of Recent Literature. Journal of Geriatric Psychiatry and Neurology, 30(5), 235–252.

Muruganantham, G. 2015. Developing of E-content package by using ADDIE model. *International Journal of Applied Research*, *1*(3), 52-54.

Navarta‐Sánchez, M. V., Ambrosio, L., Portillo, M. C., Ursúa, M. E., Senosiain, J. M., & Riverol,

M. 2020. Evaluation of a psychoeducational intervention compared with education in people with Parkinson's disease and their informal caregivers: a quasi‐experimental study. *Journal of Advanced Nursing*, *76*(10), 2719-2732.

Nor Azlin Mohd Nordin. 2013. Functional status and quality of life of community-dwelling stroke survivors and cost-effectiveness of a carer-assisted home-based therapy. PhD thesis. Universiti Kebangsaan Malaysia.

Opara, J., Małecki, A., Małecka, E., & Socha, T. (2017). Motor assessment in Parkinson`s disease. *Annals of agricultural and environmental medicine : AAEM*, *24*(3), 411–415. https://doi.org/10.5604/12321966.1232774

O. Nyumba, T., Wilson, K., Derrick, C. J., & Mukherjee, N. (2018). The use of focus group discussion methodology: Insights from two decades of application in conservation. *Methods in Ecology and evolution*, *9*(1), 20-32.

Pal, G., & Goetz, C. G. 2013. Assessing bradykinesia in parkinsonian disorders. *Frontiers in Neurology*, *4*, 54.

Poewe, W., and Mahlknecht, P. 2009. The clinical progression of Parkinson’s disease.

Parkinsonism and Related Disorders, 15(S4), S28–S32.

Rajiah, K., Maharajan, M. K., Yeen, S. J., & Lew, S. 2017. Quality of Life and Caregivers' Burden of Parkinson's Disease. *Neuroepidemiology*, *48*(3-4), 131–137. https://doi.org/10.1159/000479031

Ray Dorsey, E., Elbaz, A., Nichols, E., Abd-Allah, F., Abdelalim, A., Adsuar, J. C., Murray, C.

J.L. 2018. Global, regional, and national burden of Parkinson’s disease, 1990–2016: a systematic analysis for the Global Burden of Disease Study 2016. The Lancet Neurology, 17(11), 939–953.

Rosdinom, R., Fazli, A., Fairuz, M.A.R., Marhani, M., Hatta, S. 2011. Burden of care among caregivers to patients with Parkinson's disease: A cross-sectional study. Clinical Neurology and Neurosurgery, 113 (8), 639-643

Russell, J. D. 1974. Modular Instruction: A Guide to the Design, Selection, Utilization and Evaluation of Modular Materials.

Sidek Mohd Noah & Jamaludin Ahmad. 2005. *Pembinaan Modul: Bagaimana Membina Modul Latihan dan Modul Akademik*. Serdang: Penerbit Universiti Putra Malaysia.

Schrag, A., Selai, C., Jahanshahi, M., & Quinn, N. P. 2000. The EQ-5D—a generic quality of life measure—is a useful instrument to measure quality of life in patients with Parkinson's disease. *Journal of Neurology, Neurosurgery & Psychiatry*, *69*(1), 67-73.

Shim, V. K., Ng, C. G., & Drahman, I. (2018). Validation of the Malay Version of Zarit Burden Interview (MZBI). *Malaysian Journal of Psychiatry*, *26*(2), 3-18.

Shirley Ryan Ability Lab. 2022. Movement Disorder Society Sponsored Unified Parkinson’s Disease Rating Scale. Available at: [https://www.sralab.org/rehabilitation-](https://www.sralab.org/rehabilitation-measures/movement-disorder-society-sponsored-unified-parkinsons-disease-rating-scale) [measures/movement-disorder-society-sponsored-unified-parkinsons-disease-rating-](https://www.sralab.org/rehabilitation-measures/movement-disorder-society-sponsored-unified-parkinsons-disease-rating-scale) [scale](https://www.sralab.org/rehabilitation-measures/movement-disorder-society-sponsored-unified-parkinsons-disease-rating-scale). Accessed on: 16 Feb 2022.

Sousa, V. D., & Rojjanasrirat, W. 2011. Translation, adaptation and validation of instruments or scales for use in cross-cultural health care research: a clear and user-friendly guideline. *Journal of evaluation in clinical practice*, *17*(2), 268–274. https://doi.org/10.1111/j.1365-2753.2010.01434.x

Steffen, T., & Seney, M. 2008. Test-retest reliability and minimal detectable change on balance and ambulation tests, the 36-item short-form health survey, and the unified Parkinson disease rating scale in people with parkinsonism. *Physical therapy*, *88*(6), 733–746. <https://doi.org/10.2522/ptj.20070214>

Szende, A., Oppe, M. & Devlin, N. 2007. *EQ-5D value sets: inventory, comparative review and user guide.* Netherlands: Springer Science and Business Media.

Tuckman, B. W., & Waheed, M. A. 1981. Evaluating an individualized science programme for community college students. Journal of Research in Science Teaching, 18, 489-495.

Van de Marck, M.A., Kalf, J.G.,Stunkerboom, I.H.W.M., Nijkrake, M.J., Munneke, M., Bloom, B.R. 2009. Multidisciplinary care of patients with Parkinson’s disease. Progress in Neurology and Psychiatry, 16(2), 10–14.

Van Reenen, M. & Janssen, B. 2015. *EQ-5D-5L User Guide: Basic Information on how to use the EQ-5D-5L instrument.* Available at: <http://www.euroqol.org/about-eq-> 5d/publications/user-guide.html.

Varatharajan, S., & Chen, W. S. 2012. Reliability and validity of EQ-5D in Malaysian population. *Applied Research in Quality of Life*, *7*(2), 209-221.

Villaseñor, T., Perrin, P. B., Donovan, E. K., McKee, G. B., Henry, R. S., Dzierzewski, J. M., & Lageman, S. K. 2020. Parkinson’s family dynamics and caregiver sense of coherence: A family‐systems approach to coping in Mexico and the United States. *Aging Medicine*, *3*(4), 252-259.

Whitehead, A. L., Julious, S. A., Cooper, C. L., & Campbell, M. J. 2016. Estimating the sample size for a pilot randomised trial to minimise the overall trial sample size for the external pilot and main trial for a continuous outcome variable. *Statistical methods in medical research*, *25*(3), 1057–1073. https://doi.org/10.1177/0962280215588241

Yang, J. X., & Chen, L. 2017. Economic burden analysis of Parkinson’s disease patients in China. *Parkinson’s Disease*.

Zhao, N., Yang, Y., Zhang, L., Zhang, Q., Balbuena, L., Ungvari, G. S., Zang, Y. F., & Xiang, Y.

T. 2021. Quality of life in Parkinson's disease: A systematic review and meta-analysis of comparative studies. *CNS neuroscience & therapeutics*, *27*(3), 270–279. <https://doi.org/10.1111/cns.13549>
