# Supplementary material for: A single-blind, randomised control trial on the effectiveness of a structured multi component training module for family caregiver of persons with Parkinson’s disease: A study protocol
Source: PLoS One. 2024 Nov 11;19(11):e0309648. doi: 10.1371/journal.pone.0309648 (PMC11554143; doi:10.1371/journal.pone.0309648)
Supplement: Attachment — Submitted filename: Tabulated Response To Reviewers.docx [file pone.0309648.s003.docx]

**TABLE OF CORRECTIONS AGAINST PLOS ONE REVIEWERS’ FEEDBACK**

**Manuscript Title: A Single-Blind, Randomised Control Trial on the Effectiveness of a Structured Multi Component Training Module for Family Caregiver of Persons with Parkinson’s Disease: A Study Protocol**

**Manuscript ID: 08430283**

| **No.** | **Section** | **Comment/Feedback** | **Corrections Made/Remarks** | **Location** |
| --- | --- | --- | --- | --- |
| 1. | **General** | In general, the protocol needs to be much more precise and detailed. Using the protocol, it should be possible for a reader to be able to conduct the trial identically at another site. Terms such as **"such as"** (16 times) are not appropriate. | Details added in all sub-sections of methodology including in Spirit flowchart.  The term “such as”, and other vague statements were removed. | 1. **Page 6, line 145 to Page 24, line 581** |
| 2. | **Sample Size** | More information is needed to explain and justify the **sample size**. It seems rather optimistic by comparison with other similar trials. Was the patient population in ref 15 (Bueno) the same as proposed in this trial? Ref 16 included patients with HY stage 1-3. How was the "effect size of 0.41" derived?  If a significant clinical benefit can be shown with 36 dyads, is it appropriate to recruit another 24 merely to have 60 dyads to enable economic benefits to be assessed (or is a 40% drop out expected?)  No detail is given about the cost analysis or how the relevant data for this will be collected. | Sample Size Calculation revised. Primary outcome measure changed to MDS-UPDRS as suggested by reviewers. Effect size was derived from a past study by van der Kolk and co-authors (2019) and calculated using the formula documented in Kang (2021) for i.e. using the post intervention mean and standard deviation of MDS-UPDRS part III of the home-based and remotely supervised aerobic exercise training for PD (21·2 ±2·0) and control group (20·3 ±2.0).  Based on the new calculation, a total of 36 pairs of participant are required, i.e. 18 pairs in each group which results in a total of 72 participants .  Yes, for cost-effectiveness evaluation, it is recommended that a minimum of 30 samples for each outcome is required. Because there are patient-related and caregiver-related outcomes, recruitment of 30 pairs (i.e. 30 patients and 30 caregivers) are proposed for each group, making a total 60 pairs or 120 participants.  Method for cost and cost-effectiveness assessment are now included. Cost variables are itemized in Table 4. | 1. **Page 9, line 217 to page 10, line 238** 2. **Page 9, line 230 to Page 10, line 238** 3. **Page 20, line 458 to page 22, line 527** |
| 3. | **Study Outcomes** | There should be **only one primary outcome**. If TUG is chosen, then why would any difference not be due to general fitness secondary to the regular caregiver training rather than something specific to PD?  If UPDRS is the primary outcome, how does this impact on sample size?  Why was an outcome such as PDQ-39 or NMSQ not considered? | Thank you, reviewer. We could see now that TUG is not a good choice for primary outcome. We changed the primary outcome to MDS-UPDRS-Part III and calculated the sample size accordingly.  We decided to use EQ5D5L and added PDQ-39 as recommended. EQ5D5L is needed to the need to obtain health utility index for the QALY calculation for the cost-effectiveness analysis using ICER formula. | 1. **Page 17, line 385 to line 392** 2. **Page 18, line 407 to Page 19, line 436** |
| 4. | **Inclusion Criteria** | Why only **HY stage 1-2**? Such patients are only **minimally impaired & unlikely to show large deterioration within 6 months**, meaning it is likely to be very challenging to show benefit of intervention over control.  Is follow up long enough? | Thank you for your insight, reviewer. We initially thought of preparing caregivers of PD population with early stage of PD. However, after looking at our training module, we thought it better suit the stage 2-3 population. We changed our study population to HY stage 2-3. | 1. **Page 7, line 191** |

**Corrections addressing Reviewer #1 Comments**

| No. | Section | Comment | Action taken | Location |
| --- | --- | --- | --- | --- |
| - | **GENERAL** | This is a very important and significant study. | - Thank you reviewer! | - |
| 1. | **INTRODUCTION (Objectives)** | Under Introduction section, have an "Objectives section" to make the primary and secondary objective distinct. | A paragraph on study objectives and study hypothesis was added.   - **Main Objectives** (evaluate the effectiveness on physical function) - **Secondary Objectives** (evaluate the effectiveness on functional mobility, knowledge, burden, QOL, feasibility, cost-effectiveness, sustainability) - **Hypothesis**   Program is more effective compared to usual care | 1. **Page 5, line 133-141** |
| 2. | **METHODS (Randomisation)** | More details randomisation method  i.e. who generated the randomisation schedule? Allocation ratio, variable block randomisation etc... | Randomization method description added. Randomization was carried out by using a software by an independent researcher. Allocation of 1:1 ratio for experimental and control. Stratification variables included. | 1. **Page 10, line 255 to page 11, line 270** |
| 3. | **METHODS (Data Analysis)** | In the Data analysis, this is an RCT. Mention that results will be reported in accordance with the CONSORT 2010 guidelines. Finalization of analysis plan prior to unblinding of the data.  Briefly Mention handling of missing data? | Details of data analysis approach added.  Explanation about ‘Intention to treat’ analysis approach and how to handle missing outcome data provided. | 1. **Page 23, line 557 to page 24, line 582** 2. **Page 23, line 560** |
| 4. | **METHODS (Sample)** | Define intention to treat population. | Please accept our apology for the type error. It is intention to treat analysis, not intention to treat population. We corrected the term. |  |
| 5. | **METHODS**  **(Procedure)** | What about non-compliance or adherence, consideration of how this will be handled? | Participants with low adherence will be followed up and motivation provided to improve compliance to intervention. | 1. **Page 23, line 560** |
| 6. | **METHODS (Data Analysis)** | Any consideration of sensitivity analysis, i.e to be detailed in analysis plan, but perhaps to consider impact of non-adherence on treatment effect. | Thank you for the suggestion. We wish to not include sensitivity analysis in the protocol but will consider this valuable suggestion if our study found substantial non-adherence rate. |  |
| 7. | **METHODS** | Mentioning drop-outs due to intention to treat may not be appropriate. Intention to treat relates to including all in the analysis regardless of the randomised group and regardless of non-compliance.  Consideration of drop-outs (line 226) in studies is to make sure that the study is powered enough to ensure that you can still answer the research question, please consider revising this statement. | Statement on drop-outs is removed as this study will only utilise Intention to treat analysis and all outcome data will be made available for analysis. | **1.** **Page 9, line 235** |

**Reviewer #2 Comments**

| No. | Section | Page | Comments | Actions taken | Location |
| --- | --- | --- | --- | --- | --- |
| 1 | General comment | - | This study aimed to design a structured, multi-component training program for caregivers to implement at home for patients with PD. While the aim of the study may have its merit, there are multiple study design details need to be clarified in order to determine the novelty as well as importance of the study. | Study methodology details added. | 1. Page 6, line 145 to line 165 |
| 2 | **INTRODUCTION** | Page 2 | In the introduction, it is not clear why the authors want to focus the structured training programs for caregivers. Some discussion should be added regarding the potential advantages and disadvantages of having the caregivers to train people with PD. | Information about the importance of a comprehensive, structured program added. Statements about involving caregivers also added. | 1. Page 4, line 94 to page 5, line 123 |
| 3. | **METHODS** | Page 6,  line 157-170: | Page 6, line 157-170: the study hypothesis should be moved to the Introduction section, following research aim.  i. If the primary goal is to reduce cost, then cost-effectiveness analysis should be placed as the primary outcome. | 1. Hypothesis is moved to Introduction section  2. We wish to keep costing assessment as one of the secondary objectives | 1. Page 5, line 133-141 |
| 4. | **METHODS**  **(Inclusion Criteria)** | Page 8-9 | Why focus on patients with PD at Hoehn and Yahr stage I & II? The early stages of patients are still active and can engage in various outdoor activities. Additionally, patients with PD at this stage do not need full-time caregivers to take care of them.  Thus, the exclusion criteria of the caregiver in Table 1 seems to be awkward. Why not include patients with PD at Stage III? Patients at later stages would suffer a lot of motor and non-motor symptoms, and would thus significantly impact their ADLs and quality of life. They might also rely more on their caregivers as well as having longer time to stay at home. | Thank you for the insight, reviewer. We agree with your suggestion and have changed the study population to HY stage 2-3. | 1. **Page7, line 192** |
| 5. | **METHODS**  **(Inclusion Criteria)** |  | Since caregivers will be the primary person providing the intervention for this study, the relationship of the patients with their caregivers should be considered. Additionally, the compliance and education level of the caregivers should also be considered. | Information on the patient and caregiver relationship, caregiver education level, and occupation (income level) will be collected/identified. | 1. **Page 17, line 378 to line 382** |
| 6. | **METHODS**  **(Sample Size)** | Page 9-10 | The calculation of sample size needs to be revised. Based on my understanding of the study goal and study design, each patient with PD should be matched with 1 caregiver, so the sample size should consider as pairs instead of calculating patients and caregivers as each individual leading to a double size sample. Hence, according to the sample size calculation, 30 pairs of patient-caregiver should be recruited for each group leading to 60 pairs (60 patients and 60 caregivers) in total. | Sample size calculation revised.  30 pairs per arm, with a total of 120 participants. | 1. **Page 9, line 217 to page 10, line 238** |
| 7. | **METHODS**  **(Caregiver Training Sessions)** | Page 12: | Explain more detail about the caregiver training sessions, including the number of sessions, the duration of each session, and the way to make sure the caregivers can implement the techniques properly. | Caregiver training sessions detailed  1. A total of 7 components  2. Conducted in 3 sessions:  (1=Medical, Nursing)  (2=Diet, Speech)  (3=PT, OT and PSY)  Each component is allocated 1 hour.  Each session will last for 1-2 hours.  Conducted by experts in their respective fields. A written module and recorded training video will be provided to ease implementation of care/therapy at home. | 1. Page 11, line 277 to page 13, line 297 |
| 8. | **METHODS**  **(Control Group)** | Page 13: | What will be the treatment dosage of the control group? Will it be the same as the Experimental group? In Table 2, there seemed to be not equivalent in the treatment dosage. This will be a huge confounding factor. | Intervention for the control group described. The usual care will be kept as it occurs naturally and care components will be documented in detail for analysis and discussion. Strength and limitation of the usual care will be discussed, including recommendation for improvement if required following study completion. | 1. **Page 13, line 301 to line 312** |
| 9. | **METHODS** | Table 2 | The content that the family members going to apply is too vague.  How to make sure whether the family members know what to provide to the patient at what timing? | For therapy provision, the family caregivers will be provided with a module and logbook containing all the required tasks. Weekly phone calls will be made to follow up on any arising issue and address any concern from the caregiver. | 1. **Page 13, line 299** |
| 10. | **METHODS**  **(Primary Outcome)** | Page 14 | Why choose TUG as the primary outcome? The hypothesis put cost-effectiveness as the primary benefit of the experimental group. | We changed the primary outcome to motor function as this is more appropriate. Primary outcome measure is now MDS-UPDRS, while TUG is included in secondary outcomes. The hypothesis is revised accordingly. | 1. **Page 17, line 385 to line 392** 2. **Page 5, line 133-141** |
| 11. | **METHODS**  **(Non-motor outcome measures)** | Page 14 | The authors talked a lot about non-motor symptoms in the Introduction. However, there are very limited outcomes evaluating non-motor symptoms, except for some parts of UPDRS. I would suggest the authors add more outcomes testing non-motor symptoms for this study. | Statements about non-motor symptoms are minimized due to the study focus being motor function and mobility. |  |
| 12. | **Discussion** |  | Why establishing a protocol in Malaysia is important? Why cannot the authors use the same protocol established in other countries? Are there any specific culture-specific health care issues in Malaysia that require the conduction of this trial? | 1. **Cultural Context** 2. **Comprehensive Training Needs** 3. **No Local Data** 4. **Caregiver Burden** | **Page 25, line 597 to line 635** |
